# A Practical Treatise on Inflammation of the Uterus, Its Cervix and Appendages, and on Its Connection with Uterine Disease

**Published:** 1854-04

**Authors:** 


ART. V.—*A Practical Treatise on Inflammation of the Uterus, its cervix and appendages, and on its connection with Uterine Disease.* By JAMES HENRY BENNETT, M. D., M. R. C. P., etc., etc. Fourth American, from the third and Revised London edition. Philadelphia: Blanchard & Lea. 1853.

When we received this volume, we thought, from a casual glance at its size and appearance, that it was an abridged edition, but upon a careful comparison with the second edition upon our shelves, we found that though destitute of the thick paper, coarse type, and wide margins of the British book, it, though considerably less in size, contained much more matter than did its more assuming predecessor.

Many additions have been made. Large portions of the work have been subjected to revision, and some chapters almost re-written. Thus the second chapter of the new edition is an addition, containing a tolerably full, and very neat, description of the anatomy and physiology of the female organs of generation. The third chapter (on the frequency and importance of inflammation in uterine pathology) is much fuller than before, while the fifth chapter (considering generally the organic lesions of the cervix uteri) is almost entirely re-written.

So through the whole work we have evidence of added thought and study: of new facts obtained, and of clearer views of treatment. The work is now a standard treatise. Most of our readers are undoubtedly familiar with its previous editions, and we can only hope to call the attention of those who have not recognized these new views of uterine pathology, to their real importance.

Every book has, or should have, a thought—a dominant idea—which governs it, and guides all its reasonings to some certain conclusion with which the author is impressed, and which he seeks to impress upon his readers.

This book has *two* thoughts. One of them is the great frequency of inflammation, or other organic change in the organ itself, in the various uterine discharges and diseases. The other involves the importance of ocular demonstration by the use of the speculum.

We can have no doubt that the condition of the cervix uteri is too much overlooked in the treatment of vaginal discharges. The little pain which may accompany any extensive ulceration of the cervix, often blinds the eyes of the practitioner to its real condition. That part of the uterus is but sparsely supplied with nerves, and extensive disease there may only manifest itself by those concomitant disordered manifestations which are known alike as symptoms of leucorrhœa, menorrhagia, suppressed and retained menstruation, irritable uterus, and not unfrequently hysteria and spinal irritation. In proof of this, Dr. Bennett adduces the record of three hundred cases, accompanied by these disorders, in which the speculum revealed organic lesions of the os or cervix.

While we concede that Dr. Bennett is entirely right in his ideas of the frequency of uterine inflammation, we would object to the enthusiasm of his followers who ignore all other uterine disease, and look upon the caustic as the only remedy for all diseases of this organ. True, Dr. Bennett is not himself responsible for these extravagant views, but he has been made by many captious critics to father all the blunders of less discriminating men.

Dr. Bennett is very excusable for his penchant for the speculum. It is an abused pet of his, for which he has been made to run a muck of very bitter opposition. He has defended it most manfully, and of course cherishes the instrument for which he has withstood so virulent a crusade. It may be that he somewhat overestimates the tool, and "speculates" a trifle too freely, but he has a large capital to fall back upon.

The objections urged on the score of morality and delicacy of feeling, apply with equal force to the digital examination, while the latter is deficient in the information it conveys. Properly guarded and applied, there is really very little to shock the most fastidious in its use, while we doubt the Christian kindness of allowing disease to progress, the health to be injured, and pain to be endured, that we may spare our patient the shame of a blush, or even a salacious thought.

"To the pure all things are pure." Medicine should have dignity enough to sustain itself from any such imputations. We do not believe that male obstetricy, or male examinations of the sexual organs, when conducted in the honest effort for health and life, will injure the moral sense of any woman whose moral sense sits firmly enough upon her to be worth preserving.

For sale by Miller, Orton & Mulligan. H.

